# Spreading of α-Synuclein and Tau: A Systematic Comparison of the Mechanisms Involved

**DOI:** 10.3389/fnmol.2019.00107

**Published:** 2019-04-25

**Authors:** Eftychia Vasili, Antonio Dominguez-Meijide, Tiago Fleming Outeiro

**Affiliations:** ^1^Department of Experimental Neurodegeneration, Center for Nanoscale Microscopy and Molecular Physiology of the Brain, Center for Biostructural Imaging of Neurodegeneration, University Medical Center Goettingen, Goettingen, Germany; ^2^Max Planck Institute for Experimental Medicine, Goettingen, Germany; ^3^The Medical School, Institute of Neuroscience, Newcastle University, Newcastle Upon Tyne, United Kingdom

**Keywords:** alpha-synuclein, Parkinson's disease, tau, Alzheimer's disease, spreading

## Abstract

Alzheimer's disease (AD) and Parkinson's disease (PD) are age-associated neurodegenerative disorders characterized by the misfolding and aggregation of alpha-synuclein (aSyn) and tau, respectively. The coexistence of aSyn and tau aggregates suggests a strong overlap between tauopathies and synucleinopathies. Interestingly, misfolded forms of aSyn and tau can propagate from cell to cell, and throughout the brain, thereby templating the misfolding of native forms of the proteins. The exact mechanisms involved in the propagation of the two proteins show similarities, and are reminiscent of the spreading characteristic of prion diseases. Recently, several models were developed to study the spreading of aSyn and tau. Here, we discuss the mechanisms involved, the similarities and differences between the spreading of the two proteins and that of the prion protein, and the different cell and animal models used for studying these processes. Ultimately, a deeper understanding of the molecular mechanisms involved may lead to the identification of novel targets for therapeutic intervention in a variety of devastating neurodegenerative diseases.

## Introduction

Alzheimer's disease (AD) and Parkinson's disease (PD) are progressive, age-associated neurodegenerative disorders. Recent epidemiological studies revealed that around 50 million people worldwide are living with AD, and more than 10 million people above 60 years old with PD, respectively (Karlawish et al., 2017; Tysnes and Storstein, 2017). The prevalence of both diseases is increased in the highest age groups and the number will escalate rapidly the coming years (Karlawish et al., 2017; Tysnes and Storstein, 2017). While the clinical features are quite distinct between the two diseases, at the molecular level they are characterized by the misfolding, aggregation, and deposition of proteins in characteristic types of inclusions (Brion et al., 1985; Kosik et al., 1986; Iwai et al., 1995; Spillantini et al., 1997). Accumulation of aggregated tau is a hallmark of AD and related tauopathies and the accumulation of alpha-synuclein (aSyn) aggregates is the hallmark of PD and related synucleinopathies (Brion et al., 1985, 1986; Kosik et al., 1986; Wood et al., 1986; Wischik et al., 1988; Spillantini et al., 1997; Baba et al., 1998; Bayer et al., 1999). aSyn and tau are abundant brain proteins, both known as intrinsically disordered proteins (IDPs) with prion-like properties, as they can misfold, seed, and spread the misfolded conformation to normal monomeric forms of each protein (Uversky and Fink, 2004; Eliezer, 2009; Bartels et al., 2010; Wu and Baum, 2010; Coelho-Cerqueira et al., 2013).

Different strains of aSyn and tau display different cell binding and penetration properties, resulting in transmission of pathology between cells (Clavaguera et al., 2009; Guo and Lee, 2011; Hansen et al., 2011; Angot et al., 2012; Kfoury et al., 2012; Masuda-Suzukake et al., 2013; Wu et al., 2013; Recasens and Dehay, 2014; Sanders et al., 2014; Grozdanov and Danzer, 2018). It is currently thought that distinct protein conformations account for differences in seeding potency. Interestingly, several studies revealed the accumulation of abnormal tau aggregates in numerous cases of aSyn deposition, and vice versa (Brion et al., 1985; Kosik et al., 1986). The coexistence of aSyn and tau aggregates suggests a strong cross-talk between tauopathies and synucleinopathies, and raises the hypothesis that cross-seeding might take place, thereby contributing to disease progression (Kosik et al., 1986; Spillantini et al., 1997; Cabrales Fontela et al., 2017). Furthermore, the interaction between aSyn and tau appear to promote the oligomerization and solubility of each other *in vitro* and *in vivo*, thereby disrupting cytoskeletal organization, impairing axonal transport, and compromising synaptic organization (Masliah et al., 2001; Giasson et al., 2003; Kotzbauer et al., 2004; Bellani et al., 2010; Cabrales Fontela et al., 2017; Sotiropoulos et al., 2017; Biswas and Kalil, 2018; Ordonez et al., 2018; Prots et al., 2018; Tuerde et al., 2018; Yuan et al., 2018). However, the exact molecular mechanisms involved in the cross-talk between the two proteins, and in the propagation of pathology, are still obscure. Here, we discuss the current knowledge about the mechanisms involved in transmission of both proteins, focusing on similarities and differences between the different spreading mechanisms.

## aSyn Structure and Function

Alpha-synuclein (aSyn) is a 14.5 kDa acidic protein of 140 amino acid residues, encoded by the *SNCA* gene (Chen et al., 1995), and is strongly implicated in PD. aSyn belongs to the synuclein family, together with beta- and gamma-synuclein.

aSyn was first isolated from the synaptic vesicles and nuclei of the electric organ of *Torpedo californica* (Maroteaux et al., 1988). In 1997, aSyn was identified as the major protein component of Lewy bodies (LBs) and Lewy neurites (LNs), the pathognomonic deposits in PD (Spillantini et al., 1997). In the same year, the first point mutation in the *SNCA* gene was associated with autosomal-dominant forms of PD, demonstrating the role of genetics in the disease (Polymeropoulos et al., 1997). Furthermore, the identification of families with duplications and triplications of the *SNCA* locus confirmed that increased levels of aSyn can cause disease (Singleton et al., 2003). These findings, along with a plethora of *in vitro* and *in vivo* studies, suggest that aSyn is a central player in a group of neurodegenerative disorders known as synucleinopathies.

aSyn is classified as an intrinsically disordered protein (IDP) as it lacks defined secondary structure (Uversky, 2003, 2011a,b; Bernado et al., 2005; Breydo et al., 2012). Although the precise physiological function of aSyn is still unclear, several studies suggest that aSyn is involved in the regulation of synaptic membrane processes and in neurotransmitter release through interactions with members of the SNARE family (Tsigelny et al., 2012; Bellucci et al., 2016). Surprisingly, studies in aSyn knockout mice revealed that aSyn is not essential for synapse formation and cell survival (Bisaglia et al., 2009).

**Figure 1:**
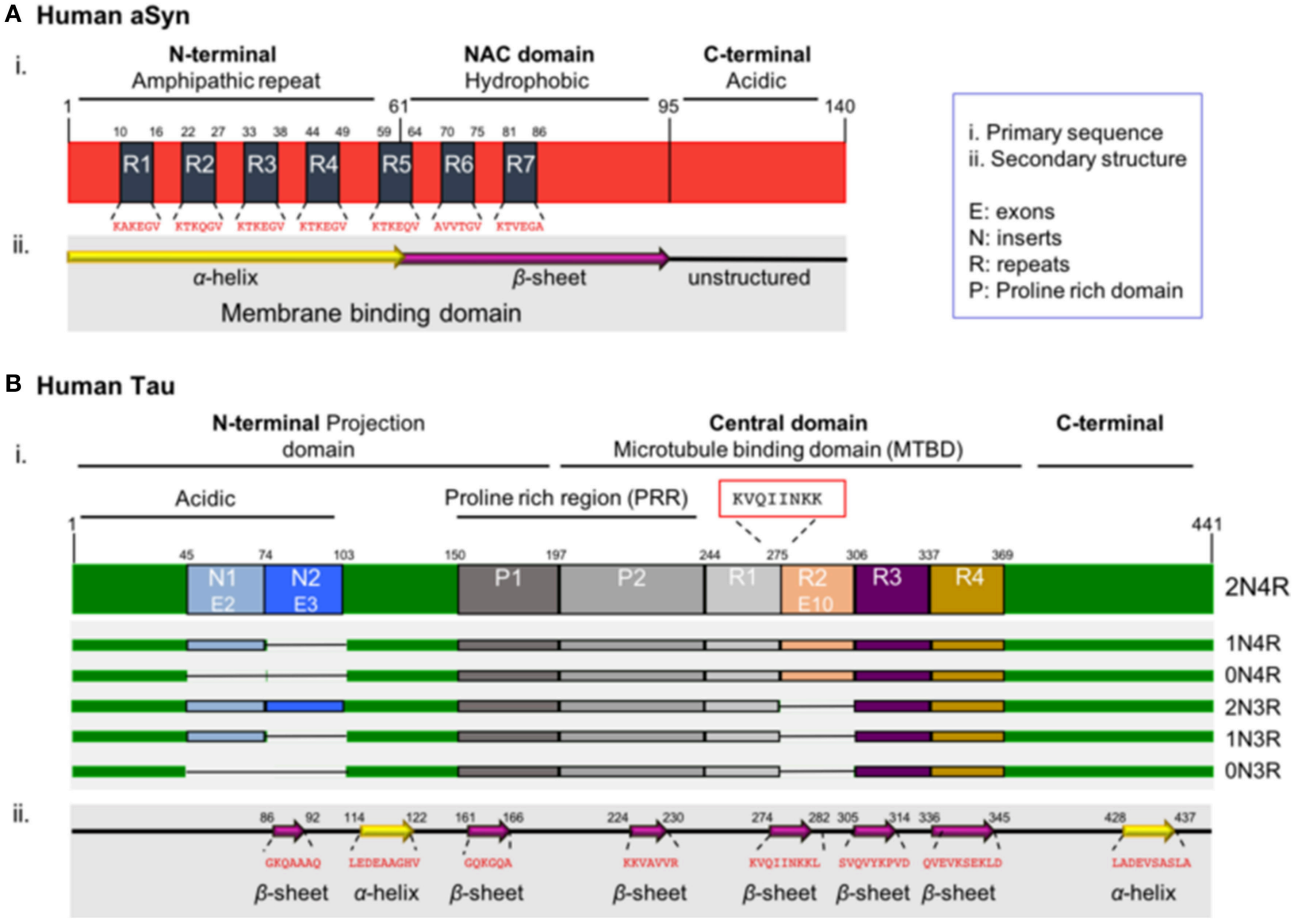
Schematic illustration of aSyn and tau proteins. **(A)** aSyn is encoded by the *SNCA* gene. The primary sequence of aSyn can be divided in three distinct domains: the amino-terminal domain (N-terminal, residues 1–60), the central domain also known as NAC domain (residues 61–95), and the carboxy-terminal domain (C-terminal domain, residues 96–140). The N-terminal domain includes four repeats (R1–R4) of the 11 amino acid alpha-helical lipid-binding motif (KTKEGV). This region has propensity to form amphipathic α-helical structures upon interacting with lipid membranes. The NAC domain (non-amyloid-β component), contains three additional repeats (R5–R7) of the lipid-binding motif, is enriched in hydrophobic residues, leading to the formation of cylindrical β-sheets and amyloid-β fibrils. Both the N-terminal and NAC domain are characterized part of the membrane binding domain. The C-terminal domain is rich in acidic residues (15 acidic amino acids: 10 Glu and 5 Asp residues) and lacks defined secondary structure. **(B)** Tau is encoded by the *MAPT* gene. Alternative splicing of the *MAPT* gene results in six isoforms known as 2N/4R, 1N/4R, 0N/4R, 2N/3R, 1N/3R, and 0N/3R, depending on the presence or absence of exon 10 (4R or 3R) and on the numbers of amino-terminal inserts (0N, 1N, and 2N) encoded by exons 2 and 3. The primary sequence of the full-length human tau isoform can be divided in the N-terminal domain also known as projection domain, the central domain which is the microtubule binding domain (MTBD) and the C-terminal tail. The N-terminal consists of the acidic part encoded by exons 2 and 3 (E2-3) called inserts 1 and 2 (N1-2), followed by the proline-rich region (PRR). The MTBD in the longest isoform contains four repeats (R1-4). The region with the strongest propensity for microtubule polymerization is the oligopeptide “KVQIINKK” (residues 274–281), located in the sub-region between the R1–R2 repeats (red box). The C-terminal tail is enriched in positively charged residues. Notably, the region with the strongest propensity for microtubule binding is located in the sub-region between the R1–R2 repeats and, more specifically, is the oligopeptide “KVQIINKK” (residues 274–281), which is included only in the 4R tau isoform, providing a stronger binding affinity when compared to the 3R isoforms (Brandt and Lee, 1993; Goode and Feinstein, 1994; Sergeant et al., 2005).

The primary sequence of aSyn can be divided in three distinct domains: the amino-terminal domain (N-terminal, residues 1–60), the central domain (residues 61–95) and the carboxy-terminal domain (C-terminal domain, residues 96–140). The N-terminal domain includes four repeats of the 11 amino acid alpha-helical lipid-binding motif (KTKEGV) (Figure 1Ai, R1–4), enabling the formation of amphipathic α-helical structures upon interaction with lipid membranes (Jao et al., 2004, 2008; Georgieva et al., 2008). The lipid composition of membranes is critical for aSyn binding. aSyn specifically prefers the binding in membranes characterized by high concentrations in cholesterol and sphingolipids, known also as lipid rafts. It seems that lipid rafts serve as a platform, which promotes aSyn binding and oligomerization (Davidson et al., 1998; Jo et al., 2000; Fortin et al., 2004; Zabrocki et al., 2008; Middleton and Rhoades, 2010; Fabelo et al., 2011; Hellstrand et al., 2013).

Importantly, all the known mutations associated with familial forms of PD are clustered within the N-terminal region of aSyn (Polymeropoulos et al., 1997; Kruger et al., 1998; Zarranz et al., 2004; Appel-Cresswell et al., 2013; Lesage et al., 2013; Proukakis et al., 2013; Pasanen et al., 2014), reinforcing the hypothesis that changes in the lipid binding domain may be linked to aSyn pathology. Interestingly, aSyn was reported to be acetylated at the N-terminus in cells, an essential modification that protects its native conformation against pathological aggregation (Iyer et al., 2016; Bu et al., 2017). The central domain, also known as NAC domain (non-amyloid-β component) (Figure 1Ai), is enriched in hydrophobic residues and is involved in the pathologic aggregation of the protein due to conformational changes (El-Agnaf et al., 1998; Giasson et al., 2001; Bellucci et al., 2012). Interestingly, one phosphorylation site is present in the NAC domain—the S87 residue. S87 phosphorylation is increased in synucleinopathies, leading to inhibition of aSyn oligomerization which influences synuclein-membrane interactions (Paleologou et al., 2010). The carboxy-terminal domain (C-terminal domain) is characterized by a non-defined structure (Bisaglia et al., 2009) and incorporates most of the posttranslational modification sites (PTMs), including the most common phosphorylation at S129 (Fujiwara et al., 2002; Oueslati, 2016). The importance of phosphorylation is emphasized by a study showing that in DLB brains, approximately 90% of insoluble aSyn is phosphorylated at S129, compared with only 4% in soluble cytosolic aSyn (Anderson et al., 2006). This suggests the implication of phosphorylation in the aggregation propensity. These PTMs may act by modulating the structure, the physiological functions and the toxicity of aSyn. Furthermore, they can modulate protein-protein interactions, interaction with metal ions (Paik et al., 1999; Brown, 2007; Bisaglia et al., 2009), including Ca^2+^ binding (Nielsen et al., 2001), polyamine complexes binding, modulation of phospholipid-binding (Paleologou et al., 2008; Visanji et al., 2011), affecting the aggregation propensity of the protein. Moreover, the region plays a protective role against aggregation, due to the presence of all the five proline (Pro) residues of the protein (Meuvis et al., 2010). Changes in the charge or the hydrophobicity by residue substitution as well as deletion of the C-terminal lead to accelerated aggregation of aSyn *in vitro* (Hoyer et al., 2004). As mentioned above, aSyn has the ability to bind to acidic membranes. This binding is mediated by the amphipathic α-helix in the N-terminal domain. Under physiological conditions aSyn exists in a dynamic equilibrium between the unfolded cytosolic and the membrane–bound state (Burre et al., 2014). In contrast, under pathological conditions, aSyn adopts a β-sheet–rich amyloid conformation, which leads to the fibril formation and subsequently aSyn deposition into LBs (Pineda and Burre, 2017). Importantly, the α-helical part is responsible for the formation of the different types of oligomers, the species currently considered to be most toxic. However, the exact nature of those toxic species remains unknown, and is still unclear whether aggregation initiates from its lipid-bound part or from the unstructured cytosolic protein (Trexler and Rhoades, 2012; Chen et al., 2015; Ghosh et al., 2015; Gallea et al., 2018).

## Tau Structure and Function

Tau was first discovered associated with microtubules, together with other microtubule-associated proteins (Weingarten et al., 1975; Kolarova et al., 2012). For this reason, it was included in the family of microtubule-associated proteins (MAPs). There are six different isoforms of tau in the central nervous system, generated from the *MAPT* gene, as a result of alternative splicing. These isoforms range from 352 to 441 amino acids (Neve et al., 1986). Each of the six tau isoforms differs in their primary structure due to the content of three (3R) or four repeats (4R) of the microtubule binding domains in the C-terminal region, in combination with the presence or absence of one (N1) or two (N2) amino acid inserts in the N-terminal part of the protein. The six isoforms are known as 0N/3R (352 residues, 60 kDa), 1N/3R (381 residues, 64 kDa), 2N/3R (410 residues, 69 kDa), 0N/4R (383 residues, 64 kDa), 1N/4R (412 residues, 69 kDa), and 2N/4R (441 residues, 74 kDa) (Figure 1 Bi), all showing higher apparent molecular masses than the predicted ones. Since the isoforms are differentially expressed in the brain during development, and stimulate microtubule assembly with different efficiencies, possibly possess particular physiological roles and implicated at different biological activities (Utton et al., 2001; Stanford et al., 2003). The shortest isoform is also known as “fetal tau isoform” because it is expressed also in the fetal brain, while all of them are detected in the human adult brain (Kosik et al., 1989; Stanford et al., 2003).

Importantly, in neurodegenerative diseases such as AD and PD, modified proportions of the different tau isoforms have been observed (Bre and Karsenti, 1990; Avila et al., 2004). Tau stabilizes the polymerization of microtubules through the three or four MTBR repeats in case of the longest isoform (Drubin and Kirschner, 1986; Maccioni et al., 1989). Under physiological conditions, in mature neurons, all tau protein is likely to be microtubule bound (Ackmann et al., 2000), and it is considered a dipole protein since the two ends of the protein have opposite charges (Sergeant et al., 2008).

The primary sequence of tau consists of the N-terminal domain, half of which is enriched in acidic residues, followed by a proline-rich region and the positively charged C-terminal tail.

Tau, like aSyn, is an intrinsically disordered protein, since it contains regions without defined secondary structure that are inserted between very short β-sheets and α-helices (Figure 1Bii). The protein can also undergo different types of PTMs like phosphorylation, ubiquitination, acetylation, glycation, methylation, truncation of the N- or C-terminal regions or nitration (Avila et al., 2004; Garcia-Sierra et al., 2008; Avila, 2009; Morris et al., 2015; Huang et al., 2016; Iqbal et al., 2016), that likely modulate its normal function and lead to pathological features. Notably, tau contains a high number of potential phosphorylation sites (80 serines/threonines and 5 tyrosines) (Grundke-Iqbal et al., 1986b; Bancher et al., 1989; Wang and Mandelkow, 2016). Most of these sites are located within the proline-rich region in close proximity to the MTBR domains and in the C-terminal tail (Figure 1Bi) (Buee et al., 2000; Sergeant et al., 2008). The phosphorylation state of the protein affects the secondary structure and, subsequently, regulates all the normal and abnormal functions like development, interaction with different protein partners such as microtubules, localization, aggregation, and spreading (Camero et al., 2014; Multhaup et al., 2015; Wang and Mandelkow, 2016). In principle, a normal and strictly controlled level of phosphorylation is required for the appropriate function of the protein, while the pathological state is characterized by hyperphosphorylation that leads the tau to lose its biological activity (Kopke et al., 1993).

The deposition of hyperphosphorylated tau in insoluble filaments in the brain is a pathological hallmark not only of AD but also of related neurodegenerative diseases, known as tauopathies, including frontotemporal dementias (FTD) like Pick's Disease and argyrophilic grain disease, progressive supranuclear palsy, and corticobasal degeneration (Grundke-Iqbal et al., 1986b). Major differences between tauopathies are the deposition of different isoforms of tau (Rademakers et al., 2004) and the occurrence of different structures of tau aggregates (Gerson et al., 2014; Dujardin et al., 2018). In AD, all the six-tau isoforms are hyperphosphorylated and aggregated into paired helical filaments (PHF) (Grundke-Iqbal et al., 1986a,b). In Pick's disease 3R isoforms are predominant, and the arrangement of tau is different than that in inclusions found in AD (Falcon et al., 2018). On the other hand, in the other tauopathies only the 4R isoform is present in the filaments (Goedert, 2015). In AD brains, the abnormally hyperphosphorylated tau is presented in the cytosol inhibiting the assembly of tubulin and disrupting microtubules. Furthermore, as a result of self-assembly is accumulated into neurofibrillary deposits in neurons and glial cells (Iqbal et al., 2010). In sporadic and familial FTD, several mutations have been identified in the tau gene. Some of these mutations are thought to disrupt the normal binding of tau to tubulin resulting in pathological deposits of hyperphosphorylated tau (Rademakers et al., 2004), or makes tau more vulnerable to self-aggregation (S422E, ΔK280, R5L, P301L, and R406W) (Haase et al., 2004; van Swieten et al., 2007; Mutreja et al., 2019). As with aSyn, it is believed that in a variety of tauopathies, the most toxic species are oligomeric, but the controversy is still not fully resolved. These oligomeric species are non-fibrillar, multimeric, soluble forms of the protein (Haase et al., 2004; Ghag et al., 2018). Examples of tauopathies where oligomers have been proposed as the toxic species include AD, corticobasal degeneration, Pick's disease, and progressive supranuclear palsy (Maeda et al., 2006; Patterson et al., 2011; Gerson et al., 2014).

## Prions and Prion-like Spreading of Pathology

Prion diseases are infectious diseases that can be transmitted horizontally between individuals of the same or even different species (Costanzo and Zurzolo, 2013; Kizhakke et al., 2017). In these diseases, PrP^C^ misfolds and converts into the pathogenic form PrP^Sc^. PrP^Sc^ then acts as a template, converting endogenous PrP^C^ into additional PrP^Sc^, thereby spreading pathology in the brain (Brandner et al., 1996). Other proteins may manifest prion-like behavior. The prion-like behavior of amyloid-β has been broadly studied (Walker et al., 2016; Ruiz-Riquelme et al., 2018; Sarnataro, 2018). Alterations in amyloid-β conformation lead to aggregation and the formation of plaques, and it has been reported that amyloid-β can reach the brain form outside the CNS (Eisele et al., 2014).

The stable propagation of different misfolded protein conformations was established as a defining feature of the prion paradigm and of the prion-like spreading of pathology (Jucker and Walker, 2013). Importantly, both aSyn and tau appear to spread in a prion-like manner (Holmes et al., 2013). However, different structural features may affect the way they propagate. Each protein has a characteristic core that undergoes conformational changes and may lead to aggregation. In particular, these are the NAC region in aSyn and the MTBR (together with the final part of the poliproline region) domain in tau (El-Agnaf et al., 1998; Ackmann et al., 2000; Giasson et al., 2001). Differences in these regions may lead to differences in the aggregated species formed. Whether aSyn and tau spreading have an infectious nature like that of the prion protein remains unclear. In general, protein infectivity depends on several factors, such as irreversibility of misfolded protein assemblies, the efficiency by which precursor polypeptides are recruited into aggregates, the clearance of the aggregates, and the efficiency of the spreading of misfolded protein proteins (Brundin et al., 2010).

### Spreading of aSyn Pathology

aSyn neuropathology typically progresses in a predictable manner throughout the brain. Post mortem analysis of human brains revealed progression of neuropathology in a series of stages (Braak et al., 2003a). Initially, the lesions start in the olfactory bulb, anterior olfactory nucleus, and dorsal motor nucleus of the vagus (Ordonez et al., 2018) in what is considered the first stage. During the second stage, pathology spreads to the lower raphe nuclei, the magnocellular portions of the reticular formation and the locus coeruleus (Prots et al., 2018). In the third stage, the pathology reaches the midbrain, affecting fundamentally the substantia nigra pars compacta (Braak et al., 2003a). Pathology spreads then to the cortex during the fourth stage. In this stage the mesocortex is affected whilst the neocortex is unaffected (Braak et al., 2003a,b). In the last two stages, pathology reaches the neocortex. Initially affecting the prefrontal neocortex and then moving to the premotor areas, the primary sensory areas and the primary motor field (Braak et al., 2003a).

aSyn can cross the blood-brain barrier (Peelaerts et al., 2015) and was shown to reach the central nervous system (CNS) after gastrointestinal administration (Holmqvist et al., 2014). It has been found in the choroid plexus, where it may be produced by the choroid cells that participate in its transport between the blood and cerebrospinal fluid (Bellani et al., 2010).

Additional studies showed the spreading of aSyn from diseased to healthy tissue. Several PD patients underwent embryonic neuronal cell transplantation developed the disease years after the surgery. The postmortem analysis of the tissue showed signs of PD, including the presence of LB and LN, in the grafted tissue. Interestingly, in these studies, the presence of cytosolic aSyn phosphorylated at S129 was also shown (Kordower et al., 2008; Li et al., 2008).

These studies, together with the Braak staging hypothesis, were considered strong evidence in favor of the prion-like spreading of aSyn pathology in the brain (Olanow and Prusiner, 2009).

Following these findings, several new studies revealed that aSyn can propagate from host to grafted tissue (Desplats et al., 2009; Angot et al., 2012; Reyes et al., 2014). A different set of studies showed how the administration of brain lysates from multiple system atrophy (MSA) patients into TgM83 mice brain leads to transmission in a way that is reminiscent of the transmission of the prion protein in chimpanzee brains in a model of Kuru (Gajdusek et al., 1966; Watts et al., 2013). This process was proposed to happen through cell-to-cell transmission following not only cell connectivity, and may reach parts of the CNS away from the injection site (Luk et al., 2012). Furthermore, 9 months after the inoculation of pathological aSyn from sarkosyl-insoluble fractions from cortical brain tissue from MSA patients, aSyn aggregates were found in the side contralateral to the administration (Bernis et al., 2015). Interestingly, in all these experiments human material leads to disease in different species. The transmission among different species is one of the main characteristics of prion proteins. Another argument in favor of the prion-like behavior of aSyn is that, under certain conditions, aSyn can assemble aberrantly forming prion strains (Guo et al., 2013; Peelaerts et al., 2015). When aSyn fibrils are inoculated in Wistar rats they act as seeds imprinting their intrinsic structures, turning monomeric aSyn into fibrils. When aSyn ribbons were injected, endogenous aSyn in Wistar rats acquired this specific conformation (Breydo et al., 2012). Furthermore, when two different strains of aSyn pre formed fibrils were inoculated in mice, endogenous aSyn acquired the structure of the strain inoculated (Peelaerts et al., 2015). Also, assemblies such as fibrils and ribbons can cross the blood-brain barrier and reach the CNS after intravenous injection (Uversky, 2011b).

**Figure 2:**
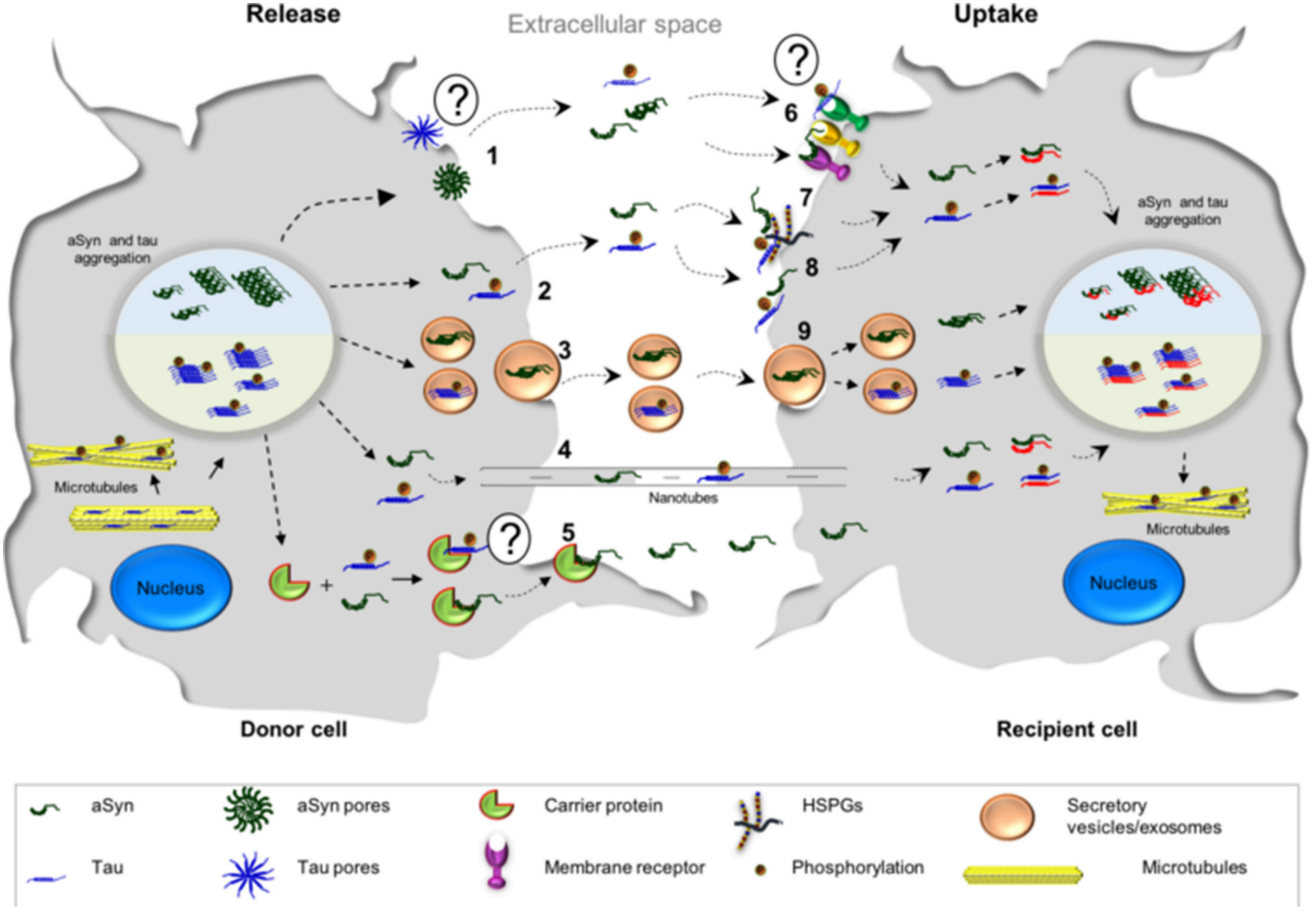
Possible mechanisms associated with the cell-to-cell transmission of aSyn and tau. The release of tau and aSyn is thought to take place via different mechanisms: (1) aSyn oligomers may form pore-like structures that penetrate the plasma membrane. These structures may act as non-selective channels, leading to the release of aSyn. At the present moment, there is less evidence in support of such mechanism for tau; (2, 8) Direct penetration of the plasma membrane may lead to protein release through passive diffusion and, consequently, passive uptake from the extracellular space, a common mechanism for both proteins; (3) aSyn and tau monomers and oligomers may be released in exosomes/secretory vesicles; (4) transmission of aSyn and tau may also occur via tunneling nanotubes which are membrane bridges between the cells composed by F-actin; (5) another possibility is that aSyn interacts with a possible carrier protein, which mediates the transfer in the plasma membrane and subsequently the release—it is still not known if tau could be released in this manner; (6) the release of both proteins may also take place from dying cells and the uptake of aSyn and tau could be mediated by cell surface receptors; (7) Heparan sulfate proteoglycans (HSPGs) may facilitate the internalization of aSyn and tau. Both proteins (monomers or fibrils) bind HSPGs at the cell surface, and then get internalized; (9) both proteins may be taken up by endocytosis.

Several mechanisms have been put forward to explain the spreading of aSyn between cells. These include membrane pores (Stockl et al., 2013), passive diffusion (Ahn et al., 2006; Grozdanov and Danzer, 2018), receptor mediated endocytosis (Mao et al., 2016), through exo- and endocytosis (Lee et al., 2005), exosomal transport (Emmanouilidou et al., 2010), tunneling nanotubes (Abounit et al., 2016a,b; Dieriks et al., 2017), and the possibility of transport through carrier proteins (Sung et al., 2001; Yang et al., 2017) (Figure 2).

aSyn is present in extracellular fluids, as it has been found in human plasma and cerebrospinal fluid and in medium from cultured human M17 neuroblastoma cell line (El-Agnaf et al., 2003). The release of aSyn has also been shown in other cell lines such as SH-SY5Y cells, H4, MES cells, and in primary neurons (Lee et al., 2005; Danzer et al., 2011; Yamada and Iwatsubo, 2018). In these cells, aSyn is released through a calcium-dependent non-conventional pathway. Treatment with compounds that interfere with the normal function of endosomes result to significant changes in the extracellular levels of aSyn. Thus, the release pathway of aSyn is dependent on the integrity of the endosomal compartment (Emmanouilidou et al., 2010; Alvarez-Erviti et al., 2011; Emmanouilidou and Vekrellis, 2016). Release can also happen through exosomes. Exosomes are vesicles of <100 nm of diameter that facilitate intercellular communication transporting proteins or RNA (Jansen et al., 2017; Mutreja and Gamblin, 2017). They can be released through budding forming a small vesicle or formed inside the multivesicular body, which fuses then with the cell membrane releasing its vesicles into the extracellular space (Beaudoin and Grondin, 1991; Denzer et al., 2000; Rustom et al., 2004). Membrane carrier proteins like the secretory carrier membrane protein 5 can also participate in the release of aSyn through exosomes (Yang et al., 2017). These release processes can happen from either the cell soma or the synaptic button, as aSyn is also transmitted trans-synaptically (Danzer et al., 2011; Freundt et al., 2012; Yamada and Iwatsubo, 2018), in processes that may or may not require axon-dendrite contacts (Freundt et al., 2012). Interestingly, mutant forms of aSyn such as the H50Q and the G51D are more prone to be released via exosomes and other types of extracellular vesicles than the wild type protein (Falcon et al., 2018). In addition, aSyn can also reach the extracellular space, not only by active mechanisms, but also passively by leakage through damaged cell membranes or by cell impairments. This process can be exacerbated by aSyn itself, as its interactions and fibrillization may disrupt cell membrane integrity (Volles and Lansbury, 2002; Chaudhary et al., 2014).

Internalization can then happen through the aforementioned mechanisms. During pinocytosis, aSyn is internalized in a dynamin-dependent process which seems to be more relevant for monomeric than for aggregated aSyn (Hansen et al., 2011). Furthermore, the endocytic process of aSyn internalization is a dynamin-dependent process, but not clathrin-dependent (Uversky, 2011a).

Tunneling nanotubes are F-actin containing membranous bridges that connect the cytoplasm of remote cells, first described in PC12 cells (Abounit and Zurzolo, 2012). Fibrillar aSyn can spread from cell to cell in a prion-like way through tunneling nanotubes by mechanisms such as intercellular trafficking of lysosomes (Abounit et al., 2016a; Dieriks et al., 2017). This happens not only among neuronal cells, but also in pericytes and astrocytes (Dieriks et al., 2017; Rostami et al., 2017), and from one type of cell to another (Sun et al., 2012).

Changes in aggregation propensity like the ones mentioned above lead to changes in the internalization and clearance of aSyn (Lee et al., 2008).

It has been proposed that aSyn can cross the cell membrane through pore-like structures such as the β-barrel voltage-dependent anion channel (Hoogerheide et al., 2017). These pores can be formed by aSyn itself in its oligomeric form, as it crosses the cell membrane leading to the formation of octameric ring structures, being the mutant A53T more prone to do it (Ma et al., 2017). The formation of these pores by aSyn is related with its binding properties to membranes and lipid layers. The association with these membranes leads to changes in membrane conductance which result to changes in its pore activity formation (Tosatto et al., 2012). The association with membranes is dependent on the presence of the KTKEGV repeat motif (Jao et al., 2004). In fact, binding of aSyn to the cell membrane causes permeabilization of the cell membrane by decreasing the lipid order (Stockl et al., 2013). This process also facilitates passive diffusion of the protein. Passive diffusion is a mechanism that allows exclusively the internalization of monomers (Lee et al., 2008). It is noteworthy that changes in the amphipatic N-terminus lead to an altered binding of aSyn to membranes. This results in abnormal vesicle interactions and changes the conformation of the vesicles, affecting aSyn spreading (Taneva et al., 2012; Dettmer et al., 2017). Out of the three known membrane proteins described to interact with aSyn, only the LAG3 and PrP^C^ mediate in its internalization through endocytosis (Chen et al., 2015; Mao et al., 2016; De Cecco and Legname, 2018). Activation of N-Methyl-D-Aspartate receptor (NMDAR) leads to the clathrin-mediated internalization of the receptor (Chen et al., 2017). Interestingly, activation of PrPc receptor by extracellular aSyn oligomers also leads to the phosphorylation of Fyn kinase activating the NMDAR receptor in a process that is independent of pore formation and impairs hippocampal long term potentiation leading to cognitive impairment (Diogenes et al., 2012; Chen et al., 2015).

### Spreading of Tau Pathology

Tau also progresses in a predictable manner throughout the CNS. In AD, tau starts its propagation in the transentorhinal region and progresses through the hippocampus, cortex and the superior temporal gyrus, finally reaching the neocortex (Uversky, 2003; Braak et al., 2006). Nonetheless, particularly in FTD cases with Pick's disease type of tau pathology, atrophy progression starts in the frontal lobe and the hippocampus, then the temporal lobe and the insula, and finally pathology reaches areas of the parietal lobe (Broe et al., 2003; Gallea et al., 2018).

In mice, tau isoforms 2N4R, 2N3R, and 0N4R were found to readily and bidirectionally cross the blood-brain barrier (Ghosh et al., 2015).

Tau propagates trans-synaptically, mostly based on connectivity and not on proximity (Ahmed et al., 2014). This propagation happens mostly through afferent connections (Iba et al., 2015) and is mediated by trans-synaptic mechanisms (Dujardin et al., 2014), where transmission was proposed to take place through exosomes (Wang et al., 2017). Although propagation happens mainly through afferent connections, small tau species can be transported anterogradely and retrogradely in neurons (Wu et al., 2013). In P301S transgenic mice, tau pre-formed fibrils lead to templated misfolding of tau in a prion-like manner along neuronal connections (Stancu et al., 2015). When injected into the brain, aggregated P301S tau present in brain homogenates from P301S mice induces the spreading of filamentous tau pathology in ALZ17 mice (Clavaguera et al., 2009). Interestingly, the P301S mutant spreads at least five times faster than the wild type (WT) tau (Kundel et al., 2018).

In normal conditions, purified human tau does not form protein assemblies (Crowther et al., 1994). Polyanionic substances, especially glycosaminoglycans such as heparin or heparan sulfate proteoglicans (HSPGs), promote tau aggregation, thereby accelerating the formation of amyloid tau fibrils (Montejo de Garcini et al., 1986; Friedhoff et al., 1998). All six tau isoforms are able to aggregate even though, *in vitro*, the 4R isoforms are more prone to aggregation than the 3R isoforms (Zhong et al., 2012). Once the protein forms assemblies, these can act as seeds for the generation of new assemblies, even when they are applied extracellularly, spreading subsequently to other cells (Goedert and Spillantini, 2017).

Tau spreads from cell to cell through several different putative mechanisms, similar to those proposed for aSyn (Guo and Lee, 2011; Tardivel et al., 2016; Katsinelos et al., 2018; Polanco et al., 2018) (Figure 2). A mechanism that was proposed to be more frequent for tau than for aSyn, is the internalization of the protein via macropinocytosis (Lee et al., 2008; Holmes et al., 2013) (Figure 2). Macropinocytosis is an endocytic process driven by actin and involves the formation of the macropinosome in response to the direct actions of cargo/receptor molecules that coordinate the activity and recruitment of specific effector molecules, and subsequently fuse with degradative compartments of the cell (Kirkham and Parton, 2005; Kerr and Teasdale, 2009). Different types of HSPGs have been described to facilitate cellular internalization of aSyn and tau *in vitro* and *in vivo* and blocking their expression diminishes the internalization of tau and aSyn monomer and aggregates (Holmes et al., 2013; Gerson et al., 2014; Dujardin et al., 2018). Oligomers and short fibrils which bind to membranes can be internalized through receptor-independent mechanisms. On the other hand, monomers, long fibrils, or long filaments, are more dependent on receptor-mediated mechanisms (Wu et al., 2013). Extracellular tau can also affect the accumulation of endogenous tau in a way that is dependent on the tau isoform found in the extracellular space. In addition, it has been shown that oligomeric 0N4R tau induces the accumulation of endogenous tau to a small extent, while oligomeric 0N3R, 1N3R, and 1N4R tau do not stimulate accumulation of intracellular tau (Swanson et al., 2017).

Detachment of tau from microtubules, e.g., due to hyperphosphorylation, increases the levels of free intracellular protein, which can then cross the membrane through translocation mechanisms (Katsinelos et al., 2018). Certain extracellular forms of tau, especially soluble forms composed mostly of monomers and small oligomers, appear to be cytotoxic through muscarinic receptor activation, involving the tissue non-specific alkaline phosphatase (Sebastian-Serrano et al., 2018). Extracellular tau, mainly truncated forms, can also contribute to synaptic dysfunction (Brandt et al., 1995; Sebastian-Serrano et al., 2018). In fact, at least 75% of tau in the synapse of AD patients is C-terminally truncated, and can be released from cortical synapses and affect its physiological role (Sokolow et al., 2015). Interestingly, these truncated forms have been reported to undergo truncation from the C-terminus to inner regions and have been linked to the pathogenesis of AD (Basurto-Islas et al., 2008; Garcia-Sierra et al., 2008). So far, two major sites of truncation have been studied, especially truncation at residues E391 and D421. Interestingly, the truncation at D421 is not only found in AD brains, but also in Pick's disease brains, where C-terminal truncated tau has been proposed to be the main isoform found in exosomes (Mena et al., 1995; Gamblin et al., 2003; Basurto-Islas et al., 2008; Mondragon-Rodriguez et al., 2008; Kanmert et al., 2015). Furthermore, overexpression of the projection domain of tau suppresses neuronal growth factor-induced neurite formation, contributing to synaptic dysfunction (Brandt et al., 1995).

## Cell Models for Studying the Spreading of aSyn and Tau

The mechanisms by which aSyn and tau spread through the central nervous system are of utmost importance to understand the progression of PD and AD, respectively. To study these mechanisms, several cell models of aSyn and tau spreading have been used in the last years.

Different cell lines have been used to study specific mechanisms underlying the spreading of aSyn and tau pathology. For instance, the mouse neuroblastoma N2a cell line has been extensively used to study cell-to-cell spreading through exosomes (Wang et al., 2017). Other cell lines, such as the HEK293, SH-SY5Y, and B103, or primary neuronal cultures, were also used to study exosomal-mediated release, but less frequently (Alvarez-Erviti et al., 2011; Polanco et al., 2016; Ngolab et al., 2017). The cell-to-cell transmission through tunneling nanotubes has been studied in the cathecolaminergic CAD cell line (Abounit et al., 2016a). The C17.2 neural precursor cell line was used to study macropinocytosis (Holmes et al., 2013) (Tables 1, 2).

**Table 1:** Cell models used to study aSyn spreading.

**Model**	**Mutation**	**Result**	**References**
SH-SY5Y	None	aSyn can be transferred from one cell to another via exosomes	Alvarez-Erviti et al., 2011
N2a; primary hippocampal neuron; N2aprnp^−/−^	None	Prp facilitates the accumulation and spreading of aSyn aggregates in N2a cells and primary hippocampal neurons	Aulic et al., 2017
HEK293T	None	Used to titer the viral vectors	Azeredo da Silveira et al., 2009
Primary cortical neurons; primary astrocytes; human Lewy body incubation	None	Quantification of aSyn uptake in neurons and astrocytes	Cavaliere et al., 2017
SH-SY5Y; primary cortical neurons; mouse cortical NSCs; aSyn expression using recombinant AdV	None	aSyn uptaken by cells; observed cell-to-cell propagation	Desplats et al., 2009
SH-SY5Y	None	Cell to cell transfer in a 3D human neuron-like cell model	Domert et al., 2016
SH-SY5Y; Tet-off system	None	aSyn is secreted through an endosomal pathway	Emmanouilidou et al., 2010
HEK293; SH-SY5Y; SKMe15: N2A/SKMe15 coculture; CM with aSyn oligos	None	aSyn can transfer between cells and seed the assembly of soluble aSyn	Hansen et al., 2011
Primary oligodendrocites; MN9D; Oli-neu; OLN93	A53T	Oligodendrocytes, Oli-neu, and OLN-93 uptake aSyn. Uptake is inhibited when clathrin heavy chain is silenced	Kisos et al., 2012
Primary cortical neurons; SH-SY5Y; KG1C; PC12	A30P; A53T	Incorporated aSyn oligomers form cytoplasmic inclusions	Konno et al., 2012
SH-SY5Y	None	aSyn is secreted through exocytosis	Lee et al., 2005
SH-SY5Y	A30P; A53T	High level of expression	Lo Bianco et al., 2002
Primary neurons; primary astrocytes; fibril administration	None	Transfer from neurons to astrocytes	Loria et al., 2017
Primary neuron, microglial and astrocyte culture; COS-7; HeLa; HEK293FT; SH-SY5Y; PFF administration; PFF with biotin; aSyn-biotin PFF administration	LAG3^−/−^	Pathologic aSyn transmission and toxicity is initiated by binding to LAG3 and that neuron-to-neuron transmission of pathological aSyn involves the endocytosis of exogenous ASyn PFF by the engagement of LAG3 on neurons	Mao et al., 2016
B103; human exosome incubation	None	Exosomes internalized by endocytosis	Ngolab et al., 2017
HEK293; infected with MSA and PD samples	A53T	Samples from MSA patients could be transmitted to cultured HEK cells but not samples from PD	Prusiner et al., 2015
OLN-93	None	Capture of monomers, oligomers and fibrils	Reyes et al., 2014
N2a; neuronal iPS; CM	None	Transfer from neuron to neuron	Reyes et al., 2015
HEK293; PC12	None	High level of expression	Yamada et al., 2004

*CM, conditioned media; MSA, Multiple System Atrophy; PFF, Pre-formed fibril*.

**Table 2:** Cell models used to study tau spreading.

**Model**	**Mutation**	**Result**	**References**
CAD; HeLa; h tau fibrils administration	None	Tau fibrils are internalized and appear inside tunneling nanotubes	Abounit et al., 2016b
Rat primary neuronal cultures; V5-Tau-LV or eGFP-LVs treatment	P301L	Cell-to-cell transfer of WT V5-Tau protein via axonal transport from the primary neurons; transferred species mainly in a dephosphorylated state	Dujardin et al., 2014
HEK293T; HEK-TREx-293; h tau transfection; sarkosyl insoluble tau and total brain lysate from TgP301S mice administration	P301S	Native Tau aggregates enter Cells through the same mechanism as recombinant Tau aggregates, consistent with macropinocytosis	Falcon et al., 2015
QBI-293; human WT, P301L, T43, t40/deltaK280, T40/P301L, and T40/R406W transfection; myc-tau and K18 fibril administration	P301L; deltaK280; R406W	Spontaneous fibril uptake is mediated by endocytosis	Guo and Lee, 2011
C17.2; HEK293; tau RD-CFP, tau RD-YFP, and FRET biosensor for tau; aSyn-488, Htt exon 1 (Q50) administration	P301L; V337M	Tau fibrils enter cells via macropinocytosis; HSPGs are receptors for cell uptake of tau and α-synuclein	Holmes et al., 2013
Mice primary neuronal cultures; HEK293T; liposome transduction of tau seeds; treatment with tau, synuclein and Htt(Q50) seeds; tau RD-CFP, tau RD-YFP, and FRET biosensor tau cell line	P301S	Seeding with tau seeds; interaction with synuclein	Holmes et al., 2014
HEK293; tau RD-CFP, tau RD-YFP, and FRET biosensor for tau	deltaK280; P301L; V337M; I227P; I308P	Repeat domain aggregates transfer between cells, induce aggregation and propagate misfolding between cells; transfer within cell medium	Kfoury et al., 2012
M1C; NB2a/d1; pRcCMV and pcDNA/V5-DEST transfection	None	Tau exon 2 insert inhibits tau secretion	Kim et al., 2010
Primary neuronal cultures; primary microglial cultures; primary astrocyte cultures; COS-7; HeLa; HEK293FT; SH-SY5Y; PFF administration; PFF with biotin; aSyn-biotin PFF administration	LAG3 -/-	Tau PFF do not bind LAG3	Mao et al., 2016
HEK293; HEK293T; tau RD-CFP, tau RD-YFP, and FRET biosensor tau cell line	P301L	Transfer of seeds through exosomes; P301L tau-containing exosome-like EVs carry tau seeds that are capable of inducing aggregation of endogenous tau after being taken up by recipient tau biosensor cells	Polanco et al., 2016
Primary neuronal cultures; mCherry-CD9-10 and Dendra2-CD9-10 transfection; exosome administration	P301L	Exchange of exosomes by interconnected neurons	Polanco et al., 2018
Primary neuronal cultures	None	Stimulation in release by neuronal activity; probably through a pre-synaptic mechanism rather than by extrusion of exosomes	Pooler et al., 2013
HEK293; tau RD-YFP, aSyn-YFP, htt exon 1 (Q25)-YFP	P301S	Seeding dependent on beta-sheet structure; tau propagates different strains; propagates to naïve cells after lysate transduction	Sanders et al., 2014
SH-SY5Y; h 3R1N, 4R1N, and HA-4R1N tau transfection; h APP-695 wild-type (WT), F690P, KM670/671NL, V717F, V717G, and APP-C99 transfection; h tau fibrils administration	F690P (for APP)	Overexpressed APP on the cell surface associates with tau fibrils and accelerates intracellular tau aggregation; transient expression of APP may increase the activity of cellular endocytosis and metabolism of APP	Takahashi et al., 2015
CAD; V5-hTau1N4R, mCherry-tubulin and GFP-actin LV infection	None	Extracellular tau species activate the formation of TNTs; tau is transported through TNTs via actin	Tardivel et al., 2016
N2A; GFP-Tau, RFP-Tau, GFP-flotillin	deltaK280	Release of seeding prone tau through exosomes; mutants for FTDP more prone; synaptic contacts are required for exosome-mediated transmission of tau	Wang et al., 2017
Mice primary neuronal cultures; HeLa; low MW aggregates administration	MAPT^−/−^	Tau aggregates are taken up in tau KO mice neurons; small tau aggregates internalized and anterogradely transported in neurons and non-neuronal cells	Wu et al., 2013
Mice primary neuronal cultures; RD-P301S YFP inoculation	P301L; MAPT^−/−^:GFP	Transfer through cell medium	Wu et al., 2016

*h, human; KO, Knockout; APP, Amyloid precursor protein; FTDP, Frontotemporal Dementia; TNT, Tunneling nanotube; WT, wild type; MW, molecular weight*.

## Animal Models for Studying the Spreading of aSyn and Tau

In recent years, animal models based in the administration of different forms of aSyn and tau in specific brain and peripheral areas have been used (Table 3). The use of animal models enables us to address specific aspects of the spreading of aSyn or tau pathology, in particular those related to neuronal connectivity, or to the spreading between different organs (Braak and Braak, 1991; Delacourte et al., 1999; Zaccai et al., 2008). For aSyn, both mice and rats have been extensively used (Hansen et al., 2011; Masuda-Suzukake et al., 2013; Holmqvist et al., 2014; Recasens and Dehay, 2014), while in the case of tau most studies employed mice (Dujardin et al., 2014).

**Table 3:** Animal models used to study aSyn spreading.

**Model**	**Site of injection**	**Material injected**	**Mutation**	**Result**	**References**
Sprague-Dawley rats	Substantia nigra	AAV2/6-haSyn, transplanted rat VM DAnergic neurons	None	Propagation from host tissue to transplanted dopaminergic neurons	Angot et al., 2012
Mice: Prnp^−/−^; prnp^+/+^	Substantia nigra	Recombinant aSyn fibrils	None	Spreading of aSyn may be facilitated by PrP	Aulic et al., 2017
Wistar rats	Substantia nigra	AAV2/6-CMV:-aSyn, -S129A-aSyn, -S129D-aSyn, -A30P-aSyn, -A30P-S129A-aSyn, -A30P-S129A-aSyn	S129A; S129D; A30P	S129A leads to the formation of smaller aggregates, S129D to larger	Azeredo da Silveira et al., 2009
Tg(SNCA)1Nbm/J	Left striatum	MSA and ILBD brain lysates inoculation Insoluble fraction of human cerebral cortices	aSyn^−/−^	Transmission of aggregates to the contralateral side 9 months after inoculation	Bernis et al., 2015
Rats: Sprague-Dawley, Wistar, Lewis; mice: C57BL/6J, SAMP8, SAMR1 *Callithrix jacchus*	Substantia nigra (rodents)	AAV2/9-p.A53T-haSyn pAAV2-CMVie/hSyn-synA53T-WPRE-pA	A53T	Spreading to striatum and throughout the whole mesencephalon in rats 16 weeks after surgery. Absence of spreading for phosphor-aSyn in rats and marmosets	Bourdenx et al., 2015
Sprague-Dawley rats	Substantia nigra	rAAV2/1-aSyn	G2019S (LRRK2); h aSyn OX	Neurodegeneration in the contralateral side	Daher et al., 2015
Sprague-Dawley rats	Substantia nigra	rAAV6-aSyn-WPRE, rAAV6-aSyn+WRPE, or rAAV-CBA-mutant aSyn	h aSyn OX	Retrograde progression of neurodegeneration	Decressac et al., 2012
(Thy1)-hαSYN transgenic mice	Hippocampus	Lentiviral GFP, stem cells, MCNSC cells	h aSyn	Transmission of aSyn from host to grafted NSCs; Inclusion body formation via cell-to-cell transmission of aSyn	Desplats et al., 2009
*Callithrix jacchus*	Substantia nigra	rAAV2/5-CBA-aSyn	A53T	Presence of aggregates in the CP 1 year after transduction in the SN	Eslamboli et al., 2007
(Thy1)-h[A30P]aSyn transgenic mice	None	None	A30P	Widespread presence of aSyn aggregates after 12 months	Freichel et al., 2007
Sprague-Dawley rats; WT C57BL/6J mice	Right cortex	Recombinant aSyn monomers, oligomers and fibrils	h aSyn	aSyn can transfer between cells and seed the assembly of soluble aSyn	Hansen et al., 2011
C57BL/6J mice; C57BL/6JOlaHsd mice	Left vagus	AAV2/6-aSyn, AAV2/6-GFP	KO of SNCA gene; h aSyn OX	Spreading from medulla oblongata to rostral brain regions	Helwig et al., 2016
Sprague-Dawley rats	Intestine wall of the stomach and duodenum	PD brain lysate, recombinant aSyn monomers, oligomers and fibrils	None	Spreading from stomach and duodenum to CNS	Holmqvist et al., 2014
BDF1 mice	Striatum	DLB brain lysate sarkosyl soluble/ insoluble fractions	h aSyn OX	Inoculation fractions of human LBD in mice leads to CNS pathology	Jones et al., 2015
Sprague-Dawley rats	Right substantia nigra	rAAV-CBA-GFP, rAAV-CBA-aSyn, rAAV-CBA-μaSyn	A53T	Overexpression of wt or mutant aSyn induce a progressive neurodegenerative pathology in the nigrostriatal DA neurons	Kirik et al., 2002
*Callithrix jacchus*	Substantia nigra	rAAV-CBA-aSyn, rAAV-CBA-μaSyn, rAAV-CBA-GFP	h aSyn OX	Spreading from medulla oblongata to rostral brain regions. Release do not enhance interneuronal hα-syn propagation	Kirik et al., 2003
A53T aSyn tg mice; Clathrin silencing	None	None	A53T	Oligodendrocytes, Oli-neu and OLN-93 uptake aSyn. Uptake is inhibited by silencing the expression of clathrin heavy chain	Kisos et al., 2012
Sprague-Dawley rats	Substantia nigra	AAV-pTR-UF12, AAV-GFP, and AAV-pSyn30	A30P	Accumulation of aSyn in SN after 12 months; presence of Lewy-like neurites in the SN and the striatum	Klein et al., 2002
Sprague-Dawley rats	Substantia nigra	AAV1/2-A53T, AAV1/2-GFP, AAV1/2-EV	A53T	Transmission to the striatum from the SN	Koprich et al., 2010
Cynomolgus macaque	Substantia nigra	AAV1/2-A53T, AAV1/2-aSyn, AAV1/2-GFP, AAV1/2-WPRE-bGH-polyA	A53T; scrambled A53T	Age-dependent increase in the accumulation of A53T; higher levels and more widespread degeneration with A53T	Koprich et al., 2016
F344 rats	Striatum and ventral mesencephalon	AAV6-aSyn, AAV6-GFP	None	Fetal DA neurons grafted into the striatum of 6-OHDA lesioned rats can retrogradely transfer h aSyn from the host into the graft	Kordower et al., 2011
Rats	Right substantia nigra	VSV-G-A30P, VSV-G-A53T, VSV-G-HWT	A30P; A53T; wt	Overexpression of wt or mutated h aSyn leads to dopamine neuronal cell death in rodents not only in the site of injection	Lo Bianco et al., 2002
TgM83 transgenic mice	Somatosensory cortex and dorsal neostriatum	M83 mice brain lysate and recombinant aSyn fibrils	A53T; aSyn^−/−^	Pathological aSyn propagated along major CNS pathways to regions far beyond injection sites	Luk et al., 2012
Sprague-Dawley rats	Above the right substantia nigra	rAAV6-aSyn, rAAV6-GFP	None	Development of degenerative changes in the nigrostriatal axons and terminals and DA release impairments	Lundblad et al., 2012
C57BL/6 mice; CD1 mice	Striatum	Recombinant aSyn fibrils	A53T; LAG3 -/-	Pathologic aSyn transmission and toxicity initiated by binding to LAG3, endocytosis of exogenous aSyn PFFs by the engagement of LAG3 on neurons	Mao et al., 2016
C57BL/6J mice	Substanstia nigra	Recombinant aSyn monomers and fibrils	None	aSyn is deposited in neurons through a prion-like mechanism or by seed-dependent aggregation by crossing the species barrier	Masuda-Suzukake et al., 2013
TgM83 transgenic mice	Intracerebral inoculation	M83 mice brain lysate	A53T; aSyn^−/−^	Data consistent with prion-like propagation of aSyn	Mougenot et al., 2012
WT C57BL/6J × DBA/2F1 mice	Hippocampus	DLB exosomes	None	Exosomes may play a role in aSyn pathogenesis, possibly through the seeding of toxic forms of aSyn	Ngolab et al., 2017
C57BL/6 mice	Right substantia nigra	rAAV2/7-haSyn	A53T	Progressive nigral dopaminergic neuron loss, presence of aSyn-rich inclusions in the surviving cell bodies	Oliveras-Salva et al., 2013
Wistar rats	Right substantia nigra and striatum	A53T α-SYN rAAV2/7, recombinant aSyn monomers, oligomers and fibrils	h aSyn OX	aSyn crosses the blood brain barrier; propagation in a stain-dependent manner	Peelaerts et al., 2015
TgM83 transgenic mice	Right parietal lobe	PD and MSA brain lysate, M83 mice brain lysate	A53T	Transmission of MSA prions requires Tg A53T aSyn mice. Unsuccessful transmit PD to TgM83+/– mice	Prusiner et al., 2015
Mice: WT C57BL/6J; C57BI6Sv129; *Macaca fascicularis*	Above the substantia nigra (mice) and motor striatum (monkeys)	PD brain lysate	aSyn^−/−^	Whether injected into the striatum or the SNpc, LB-induced degeneration was detected earlier and more extensively at the level of striatal dopaminergic axon terminals rather than SNpc cell bodies.	Recasens and Dehay, 2014
C57BL/6J mice; Sprague-Dawley rats	Cortex	Recombinant aSyn monomers, oligomers and fibrils	h aSyn OX	Transfer from host brain to grafted oligodendrocytes; oligodendrocytes take up recombinant aSyn monomers and oligomers	Reyes et al., 2014
(Thy1)-h[A30P]aSyn transgenic mice	None	None	A30P	Nutritional factors can have a significant impact on α-synucleinopathy	Rotermund et al., 2014
Sprague-Dawley rats	Intravagal	AAV2/6-aSyn	h aSyn OX	Transfer to medulla oblongata from higher brain regions	Rusconi et al., 2018
Mice: M20^+/+^; BL6C3HF1	Lateral to the lateral ventricles	Recombinant Δ71-82 and full lenght aSyn fibrils	h aSyn OX	Non-amyloidogenic Δ71-82 aSyn induce pathology. Amyloidogenic h aSyn shows limited induction of neuronal aSyn inclusions	Sacino et al., 2013
Mice: M83^+/+^; M83^+/−−;^ M20^+/+^; C3H/C57BL6 n	Biceps femoris	Recombinant Δ71-82 and full lenght aSyn fibrils	A53T; h aSyn OX	Hindlimb intramuscular injection of fibrillar aSyn lead to CNS inclusion pathology	Sacino et al., 2014b
Thy-aSyn mice	Oral administration	PD gut microbiota	aSyn OX	Microbiota are required for the motor and GI dysfunction, postnatal gut-brain signaling by microbial molecules impact neuroinflammation and αSyn aggregation	Sampson et al., 2016
(Thy1)-h[A30P]aSyn transgenic mice	None	None	A30P	Pathological aSyn species can impair synaptic plasticity	Schell et al., 2012
*Callithrix jacchus*	Caudate nucleus and putamen	Recombinant human and mouse aSyn monomers and fibrils	None	Retrograde progression of neurodegeneration	Shimozawa et al., 2017
C57BL/6	Substantia nigra	rAAV GFP and rAAV aSym	h aSyn OX	Slow disease progression that mimics human disease and allows for earlier points of characterization and/or intervention	St Martin et al., 2007
Wistar rats	Left substantia nigra	AVV2: -aSyn, A56P-aSyn, -A30P, -A30P/A56P/A76P, -EGFP	A30P; A56P; A30P/A56P/A76P	Fibrillar and prefibrillar aSyn variants secreted from rat primary cortical neurons	Taschenberger et al., 2012
C57BL/6J × CBA/ca hybrid mice	None	None	Truncated 120 aa	h aSyn120 under the control of the TH promoter led to the formation of pathological inclusions in SN and OB	Tofaris et al., 2006
Rats; mice	Substantia nigra	AAV: -aSyn, -EGFP	Truncated 120 aa	Development of inclusions in axons	Tozzi et al., 2016
Sprague-Dawley rats	Right substantia nigra	rAAV5-αsynΔ110+rAAV5-GFP, rAAV5-αsynFL+rAAV5-GFP, or rAAV5-αsynFL+rAAV5-αsynΔ110	Truncated 110 aa	Mixture of truncated and full length, lead to increased accumulation and pathological aSyn in the striatum	Ulusoy et al., 2010
Sprague-Dawley rats	Left vagus	rAAV6: -aSyn, -GFP	h aSyn OX	Spreading from medulla oblongata to rostral brain regions	Ulusoy et al., 2013
Sprague-Dawley rats	Left vagus	rAAV6: -aSyn, -GFP	h aSyn OX	Spreading from medulla oblongata to rostral brain regions	Ulusoy et al., 2015
Sprague-Dawley rats	Substantia nigra	AAV: -aSyn, -EGFP	None	DMV nerve has a role in the transmission of aSyn from the brain to peripheral tissues	Ulusoy et al., 2017
Wistar rats	Substantia nigra	rAAV: 2/1, 2/2, 2/5, 2/6.2, 2/7, 2/8, and 2/9 eGFP	None	High widespread transgene expression	Van der Perren et al., 2011
TgM83 transgenic mice	Right parietal lobe	MSA brain lysate, M83 mice brain lysates	A53T; GFAP-Luc	Lethality upon transmission to animals and similar transmission to that of kuru, CJD, and related diseases	Watts et al., 2013
Sprague-Dawley rats	Left substantia nigra	rAAV-aSyn, rAAV-EGFP	None	Progressive dopaminergic cell loss and aggregation	Yamada et al., 2004
Mice; *Macaca fascicularis*	Substantia nigra (rodents and monkeys)	Lentiviral A53T and GFP	A53T	Age-dependent increase in the accumulation of A53T; higher levels of degeneration in monkeys than in mice	Yang et al., 2015
1K and 3K mice	None	None	E35K, 346K, 361K	3K mutation causes shift from cytosolic to membrane associated aSyn	Nuber et al., 2018
M83, M47, and haSyn mice	Hippocampus and cortex	Recombinant human, mouse A53T and E36K aSyn fibrils	A53T, E46K	Spreading of inclusion pathology only in M83 mice after 4 months	Sacino et al., 2014a

*OX, overexpressing; CP, Cerebral Palsy*.

Non-human primates have already been used to study the spreading of aSyn pathology (Kirik et al., 2003; Eslamboli et al., 2007; Shimozawa et al., 2017), but have not been reported thus far for the study of tau spreading. Interestingly, a lamprey model has been used to study the effects of several tau mutations in the progression of neurodegeneration (Lee et al., 2009).

Transgenic mice expressing mutant forms of aSyn, such as A53T and A30P, have also been used, and are proving useful for studying other synucleinopathies including MSA (Giasson et al., 2002; Prusiner et al., 2015). For tau, the rTg4510 mouse line, harboring the P301L mutation, has been widely used (Barghorn et al., 2000).

Additionally, injections of viral vectors have been also extensively used to induce aSyn and tau overexpression. In the case of aSyn, several studies reported the mimicking of relevant PD features, the progressive nature, and the spreading of pathology, in mice, rats, and non-human primates (Kirik et al., 2002, 2003; St Martin et al., 2007; Low and Aebischer, 2012). In the case of tau there are also models based on the use of viral vectors (Klein et al., 2008), including models for the study of rapid tau propagation (Asai et al., 2015) (Tables 3, 4).

**Table 4:** Animal models used to study tau spreading.

**Animal model**	**Site of injection**	**Material injected**	**Mutation**	**Result**	**References**
C57BL/6J mice	Left hippocampus	Mice brain extract	P301S	Spreading through connectivity	Ahmed et al., 2014
PS19 tau mice; microglial depletion	Medial entorhinal cortex, dentate gyrus	AAV2/6-SYN1, AAV2/6-SYN1-GFP, AAV2/6-GFP. Exosomes from microglial culture, exosomes from mouse brain lysates	P301L	Participation of microglia in the spreading of tau; mediation of exosomes	Asai et al., 2015
Wistar rats	Striatum	lentiviral hTau46WT, hTauP301L and GFP	P301L	AT8 immunoreactivity extends from the CA1 (Cornus Ammonis area 1) to the cortex in rats injected with LV-hTau46WT, whereas it is restricted to the hippocampal formation in rats injected with LV-hTau46P301L, even 8 months p.i	Caillierez et al., 2013
C57BL/6J mice; ALZ17 mice	Hippocampus and overlying cerebral cortex	Mice brain homogenate	P301S	Transmission of hyperphosphorylated tau in mice transgenic for human wt tau	Clavaguera et al., 2009
ALZ17 mice; APP23 mice	Hippocampus and overlying cerebral cortex	AD, tangle only dementia, Pick's Disease, argyrophilic grain disease, progressive supranuclear palsy, and corticobasal degeneration human brain extracts	P301S; h APP	Self-propagation of tau inclusions independently of other pathological mechanisms	Clavaguera et al., 2013
C57BL/6J mice	Intraperitoneal	P301S and WT mice brainstem homogenate	P301S	Tauopathies can be seeded in the brain by tau aggregates delivered peripherally	Clavaguera et al., 2014
rTG tau EC mice	None	None	P301L	Spreading of the pathology to downstream connected neurons	de Calignon et al., 2012
Wistar rats	Striatum	lentiviral V5-hTau46WT, hTau46WT, hTauP301L, and eGFP	P301L	Spreading of Tau throughout the brain 8 months after post-injection; transmission through connected areas; trans-synaptic transmission	Dujardin et al., 2014
C57BL/6J mice	Cortex	FL-Tau-488 fibrils	None	Tau fibrils enter cells via macropinocytosis; HSPGs are receptors for cell uptake of tau and aSyn	Holmes et al., 2013
B6C3 mice; C57BL/6J mice	None	None	P301S	Tau seeding activity detected at 1.5 months, before any changes in histopathology; hyperphosphorylated tau accumulation	Holmes et al., 2014
PS19 tau mice	Locus coeruleus	Recombinant tau fibrils	P301S	The pattern of spreading did not match neurofibrillary tangles staging in h AD brains, developed tau pathology more rapidly after tau PFF injections into the LC.	Iba et al., 2015
Lamprey	Hindbrain	WT htau23 and htau24 plasmids	P301L; G272V; V337M; R406W	All mutations accelerate progression	Lee et al., 2009
Neuropsin-tTA-Tau	None	None	P301L	Spreading from the entorhinal cortex	Liu et al., 2012
Tg tau P301L mice	Hippocampus	Recombinant tau fibrils	P301L	Single injection of tau PFFs in the hippocampus or frontal cortex of young tauP301L mice acts as a seed to induce spreading of tau pathology throughout the mouse brain; neuron loss in the hippocampus	Peeraer et al., 2015
rTg4510 mice	None	None	P301L	Transfer of seeds through exosomes; P301L tau-containing exosome-like EVs carry tau seeds that are capable of inducing aggregation of endogenous tau after being taken up by recipient tau biosensor cells	Polanco et al., 2016
B6C3 mice	Hippocampus	Mice brain homogenate	P301S; h 4R1N	Spreading from the left hippocampus after 5 weeks to the entorhinal cortex, retrospenial cortex and contralateral hippocampus	Sanders et al., 2014
rTg4510 mice	Cerebral cortex	Recombinant tau short filaments	P301L	Tau aggregates taken up in tau KO mice neurons; tau aggregates internalized and anterogradely transported between brain cells	Wu et al., 2013
rTg4510 mice; neuropsin-tTA-Tau	Medial entorhinal cortex and hippocampus	AAV5 CamKII.hM3Dq-mCherry, AAV9/CamKIIa.hChR2-mCherry, AAV9/CamKIIa.hChR2-mCherry	P301L; h tau; KO	Stimulation of release and spreading by increased neuronal activity	Wu et al., 2016
JNPL3; Tg4510 mice	Intraperitoneal	Pan-tau Abs DA9, and DA31, PHF-tau CP13, and RZ3 antibodies; and 1 conformation-specific antibody MC1	P301L	Tau detected in serum	d'Abramo et al., 2016
mice JNPL3	Hippocampus	AAV5-scFv-MC1, AAV5-eGFP	P301L	Diffusion to distant areas	Vitale et al., 2018
P19 mice	Right lateral ventricle	Human antisense oligonucleotides	P301S	Decrease in human tau mRNA reverses tau seeding	DeVos et al., 2017
SHR24, SHR72 rats	Motor cortex	Sarkosyl insoluble SHR72 and SHR24 brain homogenate	Truncated aa 151-391 3R and 4R tau	Spreading only of the SHR72 4R tau variant	Levarska et al., 2013
SHR72 rats	Hippocampus	AD brain insoluble fraction, human brain extract	Truncated aa 151-391 4R tau	Exogenous human AD tau was able to spread from the area of injection and induce tau pathology	Smolek et al., 2019

## Conclusions

Tau and aSyn pathologies spread in a manner that is reminiscent of the process of prion spreading in prion diseases, whereby misfolded forms of the proteins can act as templates and induce the misfolding of normally structured proteins. The altered proteins can spread from cell to cell throughout the CNS, and possibly also between different organs through neuronal connections.

In contrast to prion diseases, in AD and PD there is still no definitive evidence for horizontal transmission of tau or aSyn pathologies, as these proteins have not been shown to be infectious. Additionally, the spreading of pathology in synucleinopathies and tauopathies seems to be slower than that observed in prion diseases, but the reason for this is still unknown (Yekhlef et al., 2003; Peden et al., 2004; Schofield et al., 2005; Desplats et al., 2009; Angot et al., 2012; Reyes et al., 2014; Mabbott, 2017).

The putative mechanisms involved in the spreading of both tau and aSyn are thought to be similar. One difference is that tau appears to have greater propensity to be internalized through macropinocytosis. Another difference is that, thus far, no putative direct receptors have been involved in mediating tau internalization, whereas for aSyn several putative “receptors” have been reported.

From an anatomical point of view, aSyn and tau appear to spread from and to different locations, in patterns that are relatively predictable (McCann et al., 2016; Hoenig et al., 2018; Schwarz et al., 2018).

In conclusion, through the use of different model systems, it is becoming evident that the spreading of aSyn and tau pathologies share similar mechanisms, and there is hope that a deeper understanding of those mechanisms may lead to the identification of novel targets for therapeutic intervention in various neurodegenerative diseases.

## Author Contributions

EV and AD-M reviewed the literature and contributed equally to the design and writing of the manuscript. TO reviewed the final manuscript.

### Conflict of Interest Statement

The authors declare that the research was conducted in the absence of any commercial or financial relationships that could be construed as a potential conflict of interest.
